# Synthesis and Antimicrobial Activity of Some New Pyrimidinone and Oxazinone Derivatives Fused with Thiophene Rings Using 2-Chloro-6-ethoxy-4-acetylpyridine as Starting Material

**DOI:** 10.3390/molecules171113642

**Published:** 2012-11-19

**Authors:** Aisha S. M. Hossan, Hanaa M. A. Abu-Melha, Mohamed A. Al-Omar, Abd El-Galil E. Amr

**Affiliations:** 1 Chemistry Department, Girls College of Science, King Khalid University, Abha 9004, Saudi Arabia; 2 Pharmaceutical Chemistry Department, College of Pharmacy, King Saud University, Riyadh 11451, Saudi Arabia; 3 Pharmaceutical Chemistry Department, Drug Exploration & Development Chair, College of Pharmacy, King Saud University, Riyadh 11451, Saudi Arabia; 4 Applied Organic Chemistry Department, National Research Center, Cairo, Dokki 12622, Egypt

**Keywords:** citrazinic acid, acryloyl candidates, oxazinone, pyrimidinone, antimicrobial agents

## Abstract

A series of pyridines, pyrimidinones, oxazinones and their derivatives were synthesized as antimicrobial agents using citrazinic acid (2,6-dihydroxyisonicotinic acid) as a starting material. α,β-Unsaturated ketones **3a**–**c** were condensed with cyanothio-acetamide in the presence of ammonium acetate to give 2-cyanopyridinethiones **4a**–**c**, which were reacted with ethyl chloroacetate to yield the corresponding cyano esters **5a**–**c**. The esters **5a**–**c** were cyclized by action of sodium methoxide to aminoesters **6a**–**c**, which were aminolyzed with ammonia to corresponding aminoamide derivatives **7a-c**. Also, the esters **6a**–**c** were hydrolyzed with NaOH to the corresponding sodium salt **8a**–**c**, which were treated with acetic anhydride to afford 2-methyloxazinones **9a**–**c**. The latter compounds were treated with ammonium acetate to afford 2-methylpyrimidinones **10a**–**c**, followed by methylation with methyl iodide to yield 2,3-dimethyl-pyrimidinones **11a**–**c**. The antimicrobial screening showed that many of these compounds have good antibacterial and antifungal activities comparable to streptomycin and fusidic acid used as reference drugs.

## 1. Introduction

In previous work, we have found that certain substituted pyridines and their derivatives showed antimicrobial, analgesic, anticonvulsant, antiparkinsonian [1,2,3,4] and antitumor activities [5,6,7]. In addition, the biological and analgesic activities of many heterocyclic compounds containing a sulfur atom have been reviewed [8,9,10]. On the other hand, thienopyrimidine and thioxopyrimidine derivatives have promising biological [11,12] and anticancer activity [13]. Recently, some new oxazinones, thienopyrimidinones and their derivatives have been synthesized as anti-inflammatory, antimicrobial and anti-HIV agents [14,15,16,17,18]. In view of these observations and in continuation of our previous work in pyridine chemistry, we have now synthesized some novel heterocyclic compounds containing the thieno[2,3-*b*]pyridine moiety fused with a pyridine, oxazinone, or pyrimidinone, nucleus and tested their antimicrobial activities.

## 2. Results and Discussion

### 2.1. Synthesis

**Scheme 1:**
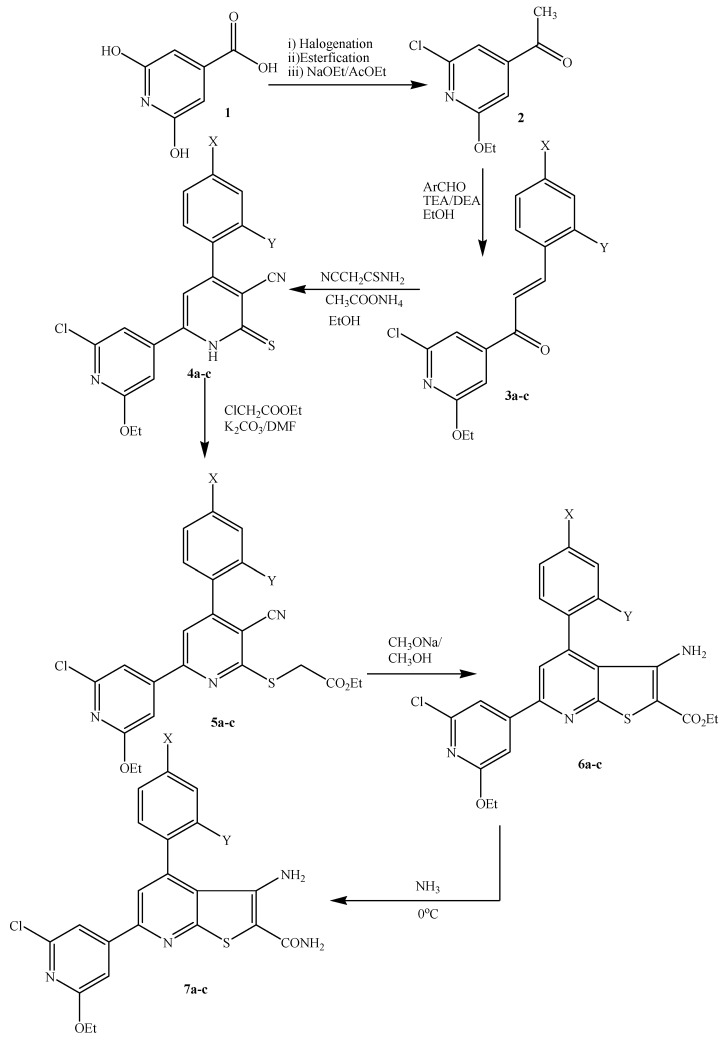
Synthetic Pathway for Compound **3**–**7**.

The starting materials **3a**–**c** (Table 1) were prepared from 2,6-dihydroxyisonicotinic acid (**1**) via the corresponding 2-chloro-6-ethoxy-4-acetylpyridine **2** according to literature methods [1,19]. Acryloyl derivatives **3a**–**c** were condensed with 2-cyanothioacetamide in the presence of ammonium acetate to give the corresponding cyanopyridine thione derivatives **4a**–**c** (Table 1). Treatment of **4a**–**c** with ethyl chloroacetate in the presence of anhydrous K_2_CO_3_ gave the corresponding ethyl ester derivative **5a**–**c** (Table 1), which were cyclized by sodium methoxide in methanol to give the amino ester derivatives **6a**–**c** (Table 1). Aminolysis of compounds **6a**–**c** by action of ammonia gas afforded the corresponding aminoamide derivatives **7a**–**c** (Scheme 1, Table 1). The IR spectra of **6a**–**c** showed the absence of ν (C≡N) for **5a**–**c** and the presence of broad band corresponding to ν (NH_2_). Also, the IR spectra of **7a**–**c** showed the absence of ν(C=O, ester) for **6a**–**c** and the presence of a broad band corresponding to ν(NH_2_).

**Scheme 2:**
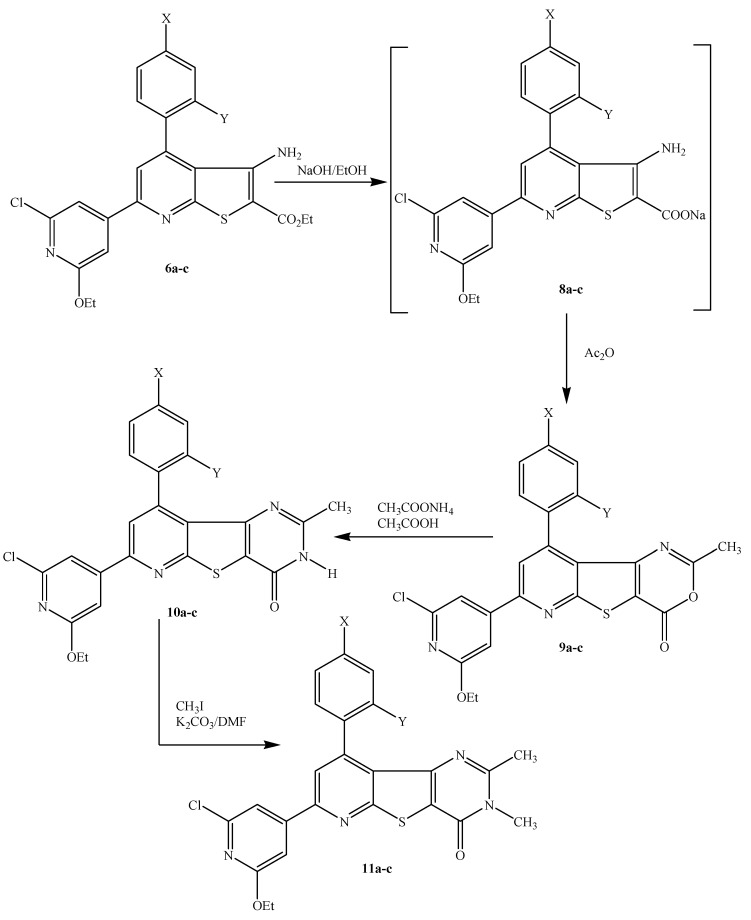
Synthetic Pathway for Compound **9**–**11**.

Compounds **6a**–**c** were hydrolyzed by refluxing with ethanolic sodium hydroxide (NaOH) to the corresponding sodium salts **8a**–**c**, which was treated *in situ* with refluxing acetic anhydride to give the corresponding oxazinone derivatives **9a**–**c** (Table 2). Reaction of **9a**–**c** with ammonium acetate in refluxing acetic acid afforded the corresponding pyrimidinone derivatives **10a**–**c** (Table 2), which were treated with methyl iodide in *N,N-*dimethylformamide in the presence of anhydrous K_2_CO_3_ to yield the corresponding 3-methyl-pyrimidinone derivatives **11a**–**c** (Scheme 2, Table 2).

### 2.2. Antimicrobial Activity 

The antimicrobial activities of some of the synthesized compounds were determined by the agar diffusion method as recommended by the National Committee for Clinical Laboratory Standards (NCCLS) [20]. The compounds were evaluated for antimicrobial activity against bacteria, *viz*. *Streptomyces* sp., *Bacillus subtilis*, *Streptococcus lactis*, *Escherichia coli*, and *Pseudomonas* sp. and antifungal activity against various fungi, *viz*. *Aspergillus niger*, *Penicillium* sp and yeast *Candida albican* and *Rhodotorula ingeniosa*.

The concentrations of the tested compounds (10 µg/mL) were used according to a modified Kirby-Bauer’s disk diffusion method. The sterile discs were impregnated with 10 µg/disc of the tested compound. Each tested compound was performed in triplicate. The solvent DMSO was used as a negative control and streptomycin/fusidic acid were used as standard calculated average diameters (for triplicates) of the zone of inhibition (in mm) for tested samples with that produced by the standard drugs. Four of the synthesized compounds **5a**, **7b**, **9b** and **10b** exhibited potent antibacterial and antifungal bioactivity compared with the standard drug used. The other tested compounds were found to exhibit a moderate to low antibacterial activity (Table 3).

On the other hand, when different concentrations of compound **9a** were used, it was exhibited a moderate antibacterial activity, but it exhibited very good antibacterial activity at higher concentrations (3× and 4×) (Table 4), while different concentrations of compounds **5a** and **10a** exhibited very good antifungal activities (2× and 3×) (Table 5).

## 3. Experimental 

### 3.1. Chemistry

Melting points were measured using Electrothermal 9100 digital melting point apparatus (Electrothermal, Essex, UK) and are uncorrected. IR spectra were recorded on a Perkin-Elmer 1600 FTIR (Perkin-Elmer, Downers Grove, IL, USA) in KBr discs. ^1^H- and ^13^C-NMR spectra were measured on a Jeol 5000 MHz spectrometer (Jeol, Tokyo, Japan) in DMSO-*d*_6_, and chemical shifts were recorded in δ ppm relative to the internal standard TMS. The Mass spectra were run at 70 eV with a Finnigan SSQ 7000 spectrometer (Madison, WI, USA) using EI and the values of *m/z* are indicated in Dalton. Elemental analyses were performed on a Perkin-Elmer 2400 analyzer (Perkin-Elmer) and were found within ±0.4% of the theoretical values. All reactions were followed by *TLC* (Silica gel, Aluminum Sheets 60 F_254_, Merck, Darmstadt, Germany). Starting material **2** was prepared from citrazinic acid (**1**) according to published procedures [1,19]. Antimicrobial screening was carried out in Department of Microbial Chemistry, National Research Center, Cairo, Egypt.

*1-(2-Chloro-6-ethoxypyridin-4-yl)-3-(substituted phenyl)prop-2-en-1-ones* **3a**–**c**. A mixture of 2-chloro-6-ethoxy-4-acetylpyridine (**2**) [19] (1 mmol) and an aromatic aldehyde, namely, 4-flouro-, 4-chloro- or 2,4-dichlorobenzaldehyde (1 mmol) in absolute ethanol (30 mL) was refluxed in the presence of a mixture of TEA/DEA (3 mL, 1:1 v:v) for 6 h. The reaction mixture was concentrated under reduced pressure, the obtained solid was filtered off, washed with ether, dried and crystallized from the proper solvents to afford the corresponding acryloyl derivatives **3a**–**c**, respectively.

*1-(2-Chloro-6-ethoxypyridin-4-yl)-3-(4-fluorophenyl)prop-2-en-1-one* (**3a**). IR (KBr, cm^−1^): ν 1679 (C=O), 1607 (C=C); ^1^H-NMR: δ 1.32 (t, 3H, CH_3_, *J* = 6.95 Hz), 3.81 (q, 2H, CH_2_, *J* = 6.95 Hz), 6.65 (d, 1H, CH-olefinic-H, *J* = 14.60 Hz), 6.98 (d, 1H, CH-olefinic-H, *J* = 14.60 Hz), 7.28–7.96 (m, 6H, 4 Ph-H + 2 pyr-H); ^13^C-NMR: 13.68, 64.32, 104.95, 109.56, 114.72, 121.30, 129.86, 130.05, 144.65, 145.84, 146.50, 160.95, 164.96, 186.50; MS, *m/z* (%): 306 (M^+^, 15), 184 (100); Elemental analysis for C_16_H_13_ClFNO_2_ (305.73): calcd.: C, 62.86; H, 4.29; Cl, 11.60; N, 4.58. found: C, 62.80; H, 4.26; Cl, 11.55; N, 4.52.

*1-(2-Chloro-6-ethoxypyridin-4-yl)-3-(4-chlorophenyl)prop-2-en-1-one* (**3b**). IR (KBr, cm^−1^): ν 1682 (C=O), 1610 (C=C); ^1^H-NMR: δ 1.33 (t, 3H, CH_3_, *J* = 6.95 Hz), 3.92 (q, 2H, CH_2_, *J* = 6.95 Hz), 6.58 (d, 1H, CH-olefinic-H, *J* = 14.60 Hz), 7.05 (d, 1H, CH-olefinic-H, *J* = 14.60 Hz), 7.12–7.88 (m, 6H, 4 Ph-H + 2 pyr-H); ^13^C-NMR: 13.86, 64.26, 105.78, 109.62, 121.12, 126.86, 128.25, 132.85, 132.96, 144.68, 145.78, 146.65, 164.84, 186.86; MS, *m/z* (%): 322 (M^+^, 8), 165 (100); Elemental analysis for C_16_H_13_Cl_2_NO_2_ (322.18): calcd.: C, 59.65; H, 4.07; Cl, 22.01; N, 4.35. found: C, 59.60; H, 4.00; Cl, 21.96; N, 4.30.

*1-(2-Chloro-6-ethoxypyridin-4-yl)-3-(2,4-dichlorophenyl)prop-2-en-1-one* (**3c**). IR (KBr, cm^−1^): ν 1678 (C=O), 1612 (C=C); ^1^H-NMR: δ 1.28 (t, 3H, CH_3_, *J* = 6.95 Hz), 3.86 (q, 2H, CH_2_, *J* = 6.95 Hz), 6.46 (d, 1H, CH-olefinic-H, *J* = 14.60 Hz), 7.10 (d, 1H, CH-olefinic-H, *J* = 14.60 Hz), 7.25–7.76 (m, 5H, 3 Ph-H + 2 pyr-H); ^13^C-NMR: 13.92, 64.30, 105.96, 109.46, 121.21, 125.69, 128.78, 129.56, 130.85, 132.05, 133.65, 144.86, 145.88, 146.54, 164.78, 187.05; MS, *m/z* (%): 356 [M^+^,10], 199 [100, base peak]; Elemental analysis for C_16_H_1_2Cl_3_NO2 (356.63): calcd.: C, 53.89; H, 3.39; Cl, 29.82; N, 3.93. found: C, 53.83; H, 3.34; Cl, 29.80; N, 3.88.

*6-(2-Chloro-6-ethoxypyridin-4-yl)-4-(substituted phenyl)-1,2-dihydro-2-thioxopyridine-3-carbonitriles* **4a**–**c**. A mixture of **3a**–**c** (1 mmol), 2-cyanothioacetamide (0.10 g, 1 mmol) and ammonium acetate (0.6 g, 8 mmol) in absolute ethanol (30 mL) was refluxed for 5 h. After cooling, the formed product was collected by filtration, washed with ethanol, dried and crystallized from the proper solvents to give the corresponding thioxopyridine derivatives **4a**–**c**, respectively. 

*6-(2-Chloro-6-ethoxypyridin-4-yl)-4-(4-fluorophenyl)-1,2-dihydro-2-thioxopyridine-3-carbonitrile* (**4a**). IR (KBr, cm^−1^): ν 3330 (NH), 2210 (CN), 1218 (C=S); ^1^H-NMR: δ 1.30 (t, 3H, CH_3_, *J* = 6.95 Hz), 3.90 (q, 2H, CH_2_, *J* = 6.95 Hz), 6.95–7.78 (m, 6H, 4 Ph-H + 2 pyr-H), 8.46 (s, 1H, pyr-5'-H), 9.24 (s, 1H, NH exchangeable with D_2_O); ^13^C-NMR: 13.66, 63.98, 103.66, 103.88, 107.89, 108.55, 114.58, 116.02, 127.50, 128.04, 145.48, 148.60, 160.56, 161.76, 164.65, 167.47, 168.05; MS, *m/z* (%): 386 [M^+^,24], 135 [100, base peak]; Elemental analysis for C_19_H_13_ClFN_3_OS (385.84): calcd.: C, 59.14; H, 3.40; Cl, 9.19; N, 10.89; S, 8.31. found: C, 59.10; H, 3.35; Cl, 9.14; N, 10.85; S, 8.28.

*6-(2-Chloro-6-ethoxypyridin-4-yl)-4-(4-chlorophenyl)-1,2-dihydro-2-thioxopyridine-3-carbonitrile* (**4b**). IR (KBr, cm^−1^): ν 3356 (NH), 2215 (CN), 1210 (C=S); ^1^H-NMR: δ 1.34 (t, 3H, CH_3_, *J* = 6.95 Hz), 3.86 (q, 2H, CH_2_, *J* = 6.95 Hz), 7.12–7.80 (m, 6H, 4 Ph-H + 2 pyr-H), 8.52 (s, 1H, pyr-5'-H), 9.18 (s, 1H, NH exchangeable with D_2_O); ^13^C-NMR: 13.92, 64.12, 103.96, 104.04, 108.14, 108.86, 115.82, 127.66, 128.10, 129.68, 132.67, 145.56, 148.72, 160.77, 164.58, 167.55, 167.86; MS, *m/z* (%): 402 [M^+^,32], 211 [100, base peak]; Elemental analysis for C_19_H_13_Cl_2_N_3_OS (402.29): calcd.: C, 56.73; H, 3.26; Cl, 17.63; N, 10.45; S, 7.97. found: C, 56.68; H, 3.20; Cl, 17.60; N, 10.40; S, 7.92.

*6-(2-Chloro-6-ethoxypyridin-4-yl)-4-(2,4-dichlorophenyl)-1,2-dihydro-2-thioxopyridine-3-carbonitrile* (**4c**). ν 3348 (NH), 2218 (CN), 1212 (C=S); ^1^H-NMR: δ 1.32 (t, 3H, CH_3_, *J* = 6.95 Hz), 3.78 (q, 2H, CH_2_, *J* = 6.95 Hz), 6.98–7.68 (m, 5H, 3 Ph-H + 2 pyr-H), 8.64 (s, 1H, pyr-5'-H), 9.34 (s, 1H, NH exchangeable with D_2_O); ^13^C-NMR: 14.14, 64.18, 103.88, 104.08, 108.22, 108.92, 115.76, 125.98, 128.56, 129.16, 131.86, 132.15, 134.86, 145.64, 148.80, 161.24, 164.32, 167.45, 168.18; MS, *m/z* (%): 436 [M^+^,14], 279 [100, base peak]; Elemental analysis for C_19_H_12_Cl_3_N_3_OS (436.74): calcd.: C, 52.25; H, 2.77; Cl, 24.35; N, 9.62; S, 7.34. found: C, 52.20; H, 2.71; Cl, 24.30; N, 9.57; S, 7.28.

*Ethyl 2-(6-(2-chloro-6-ethoxypyridin-4-yl)-3-cyano-4-(substituted phenyl)pyridin-2-ylthio)acetates* **5a**–**c***.* To a mixture of **4a**–**c** (1 mmol) and anhydrous K_2_CO_3_ (0.18 g, 1 mmol) in *N*-dimethylformamide (25 mL) was stirred at room temperature for 2 h, ethyl chloroacetate (0.18 g, 1.5 mmol) was added with stirring. The reaction mixture was heated at 60 °C for 2 h and after cooling poured into ice. The solid formed was collected by filtration, washed with water, dried and crystallized from the proper solvents to afford the corresponding pyridinethioacetate derivatives **5a**–**c**, respectively.

*Ethyl 2-(6-(2-chloro-6-ethoxypyridin-4-yl)-3-cyano-4-(4-fluorophenyl)pyridin-2-ylthio)acetate* (**5a**). IR (KBr, cm^−1^): ν 2219 (CN), 1735 (C=O, ester); ^1^H-NMR: δ 1.28, 1.32 (2t, 6H, 2 CH_3_), 3.68, 3.86 (2q, 4H, 2 CH_2_), 4.38 (s, 2H, S–CH_2_), 7.16–7.82 (m, 6H, 4 Ph-H + 2 pyr-H), 8.18 (s, 1H, pyr-5'-H); ^13^C-NMR: 13.65, 14.05, 32.04, 59.86, 64.08, 101.36, 101.57, 102.85, 115.02, 116.75, 117.02, 128.74, 132.58, 145.65, 151.56, 153.65, 157.08, 162.15, 163.56, 163.94, 168.90; MS, *m/z* (%): 472 [M^+^,12], 426 [100, base peak]; Elemental analysis for C_23_H_19_ClFN_3_O_3_S (471.93): calcd.: C, 58.54; H, 4.06; Cl, 7.51; N, 8.90; 17; S, 6.79. found: C, 58.48; H, 4.00; Cl, 7.45; N, 8.84; 17; S, 6.72.

*Ethyl 2-(6-(2-chloro-6-ethoxypyridin-4-yl)-3-cyano-4-(4-chlorophenyl)pyridin-2-ylthio)acetate* (**5b**). IR (KBr, cm^−1^): ν 2222 (CN), 1732 (C=O, ester); ^1^H-NMR: δ 1.29, 1.32 (2t, 6H, 2 CH_3_), 3.56, 3.84 (2q, 4H, 2 CH_2_), 4.42 (s, 2H, S–CH_2_), 7.10–7.72 (m, 6H, 4 Ph-H + 2 pyr-H), 8.64 (s, 1H, pyr-5'-H); ^13^C-NMR: 13.78, 14.15, 32.18, 60.05, 64.18, 101.48, 101.68, 102.74, 116.88, 117.02, 127.54, 128.12, 129.57, 133.45, 145.56, 150.96, 153.64, 157.18, 163.72, 164.05, 170.04; MS, *m/z* (%): 488 [M^+^,32], 120 [100, base peak]; Elemental analysis for C_23_H_19_Cl_2_N_3_O_3_S (488.38): calcd.: C, 56.56; H, 3.92; Cl, 14.52; N, 8.60; S, 6.57. found: C, 56.50; H, 3.88; Cl, 14.47; N, 8.55; S, 6.51.

*Ethyl 2-(6-(2-chloro-6-ethoxypyridin-4-yl)-3-cyano-4-(2,4-dichlorophenyl)pyridin-2-ylthio)acetate* (**5c**). IR (KBr, cm^−1^): ν 2218 (CN), 1735 (C=O, ester); ^1^H-NMR: δ 1.26, 1.30 (2t, 6H, 2 CH_3_), 3.58, 3.78 (2q, 4H, 2 CH_2_), 4.36 (s, 2H, S–CH_2_), 7.12–7.65 (m, 5H, 3 Ph-H + 2 pyr-H), 8.56 (s, 1H, pyr-5'-H); ^13^C-NMR: 13.84, 14.18, 32.18, 59.92, 64.18, 100.98, 101.59, 102.66, 116.82, 117.06, 125.86, 128.48, 129.24, 131.92, 132.24, 134.74, 145.58, 151.08, 153.72, 157.22, 163.88, 164.15, 168.84; MS, *m/z* (%): 523 [M^+^,8], 247 [100, base peak]; Elemental analysis for C_23_H_18_Cl_3_N_3_O_3_S (522.83): C, 52.84; H, 3.47; Cl, 20.34; N, 8.04; S, 6.13. found: C, 52.78; H, 3.40; Cl, 20.28; N, 8.00; S, 6.07.

*Ethyl 3-amino-6-(2-chloro-6-ethoxypyridin-4-yl)-4-(substituted phenyl)thieno[2,3-b]pyridine-2-carboxylates* **6a**–**c**. A mixture of **5a**–**c** (1 mmol) in sodium methoxide solution (20 mL, 2%) was refluxed for 1 h on a water bath at 70 °C with stirring. The reaction mixture was evaporated under reduced pressure, the obtained residue was dissolved in CH_2_Cl_2_, washed with H_2_O, 10 mL 1 N HCl and then with water. The solvent was dried over anhydrous CaCl_2_, evaporated under reduced pressure, and the obtained product was crystallized to afford from the proper solvents to afford the corresponding ethyl thienopyridinecarboxylates **6a**–**c**, respectively. 

*Ethyl 3-amino-6-(2-chloro-6-ethoxypyridin-4-yl)-4-(4-fluorophenyl)thieno[2,3-b]pyridine-2-carboxyl-ate* (**6a**). IR (KBr, cm^−1^): ν 3443 (NH_2_), 1742 (C=O, ester); ^1^H-NMR: δ 1.30, 1.34 (2t, 6H, 2 CH_3_), 3.72, 4.06 (2q, 4H, 2 CH_2_), 4.36 (s, 2H, NH_2_ exchangeable with D_2_O), 7.24–7.75 (m, 6H, 4 Ph-H + 2 pyr-H), 8.35 (s, 1H, pyr-5'-H); ^13^C-NMR: 13.95, 14.16, 60.24, 64.18, 101.58, 103.02, 115.16, 118.35, 120.76, 122.15, 128.66, 132.64, 134.12, 145.72, 149.65, 151.64, 154.57, 155.75, 160.12, 162.65, 164.12; MS, *m/z* (%): 472 [M^+^,26], 317 [100, base peak]; Elemental analysis for C_23_H_19_ClFN_3_O_3_S (471.93): calcd.: C, 58.54; H, 4.06; Cl, 7.51; N, 8.90; S, 6.79. found: C, 58.48; H, 4.00; Cl, 7.45; N, 8.86; S, 6.71.

*Ethyl 3-amino-6-(2-chloro-6-ethoxypyridin-4-yl)-4-(4-chlorophenyl)thieno[2,3-b]pyridine-2-carboxyl-ate* (**6b**). IR (KBr, cm^−1^): ν 3452 (NH_2_), 1737 (C=O, ester); ^1^H-NMR: δ 1.26, 1.31 (2t, 6H, 2 CH_3_), 3.78, 4.10 (2q, 4H, 2 CH_2_), 4.48 (s, 2H, NH_2_ exchangeable with D_2_O), 7.24–7.82 (m, 6H, 4 Ph-H + 2 pyr-H), 8.72 (s, 1H, pyr-5'-H); ^13^C-NMR: 14.08, 14.25, 60.15, 64.10, 100.42, 103.64, 118.05, 121.16, 122.25, 127.66, 128.44, 133.45, 133.95, 134.50, 146.02, 149.75, 151.18, 154.65, 156.05, 159.64, 164.15; MS, *m/z* (%): 488 [M^+^,8], 332 [100, base peak]; Elemental analysis for C_23_H_19_Cl_2_N_3_O_3_S (488.38): calcd.: C, 56.56; H, 3.92; Cl, 14.52; N, 8.60; S, 6.57. found: C, 56.50; H, 3.88; Cl, 14.46; N, 8.55; S, 6.50.

*Ethyl 3-amino-6-(2-chloro-6-ethoxypyridin-4-yl)-4-(2,4-dichlorophenyl)thieno[2,3-b]pyridine-2-carboxylate* (**6c**). IR (KBr, cm^−1^): ν 3456 (NH_2_), 1735 (C=O, ester); ^1^H-NMR: δ 1.30, 1.33 (2t, 6H, 2 CH_3_), 3.82, 4.15 (2q, 4H, 2 CH_2_), 4.56 (s, 2H, NH_2_ exchangeable with D_2_O), 7.08–7.68 (m, 5H, 3 Ph-H + 2 pyr-H), 8.62 (s, 1H, pyr-5'-H); ^13^C-NMR: 14.12, 14.26, 59.98, 64.32, 100.86, 102.72, 118.02, 121.06, 122.00, 126.16, 128.87, 129.36, 132.18, 134.05, 135.44, 136.74, 145.64, 149.85, 151.38, 154.72, 155.43, 160.04, 164.25; MS, *m/z* (%): 523 [M^+^,6], 177 [100, base peak]; Elemental analysis for C_23_H_18_Cl_3_N_3_O_3_S (522.83): calcd.: C, 52.84; H, 3.47; Cl, 20.34; N, 8.04; S, 6.13. found: C, 52.77; H, 3.42; Cl, 20.30; N, 7.97; S, 6.08.

*3-Amino-6-(2-chloro-6-ethoxypyridin-4-yl)-4-(substituted-phenyl)thieno[2,3-b]pyridine-2-carbox-amides* **7a**–**c**. A current of ammonia gas was passed through a suspension of **6a**–**c** (1 mmol) in absolute ethanol (100 mL), at 0 °C till saturation. The reaction mixture was left overnight at −4 °C, evaporated under reduced pressure, the residue obtained was triturated with *n*-hexane, the formed solid was filtered off, washed with water and crystallized from the proper solvents to give the corresponding thienopyridine carboxamides **7a**–**c**, respectively.

*3-Amino-6-(2-chloro-6-ethoxypyridin-4-yl)-4-(4-fluorophenyl)thieno[2,3-b]pyridine-2-carboxamide* (**7a**). IR (KBr, cm^−1^): ν 3460–3380 (NH_2_), 1675 (C=O, amide); ^1^H-NMR: δ 1.32 (t, 3H, CH_3_), 3.85 (q, 2H, CH_2_), 4.46, 6.85 (2s, 4H, 2 NH_2_ exchangeable with D_2_O), 7.12–7.68 (m, 6H, 4 Ph-H + 2 pyr-H), 8.56 (s, 1H, pyr-5'-H); ^13^C-NMR: 14.06, 64.28, 100.96, 102.22, 115.36, 120.82, 122.45, 128.37, 128.46, 132.84, 137.15, 145.82, 149.65, 152.00, 154.74, 157.75, 161.55, 162.76, 164.30; MS, *m/z* (%): 443 [M^+^,8], 332 [100, base peak]; Elemental analysis for C_21_H_16_ClFN_4_O_2_S (442.89): calcd.: C, 56.95; H, 3.64; Cl, 8.00; N, 12.65; S, 7.24. found: C, 56.90; H, 3.60; Cl, 7.940; N, 12.60; S, 7.19.

*3-Amino-6-(2-chloro-6-ethoxypyridin-4-yl)-4-(4-chlorophenyl)thieno[2,3-b]pyridine-2-carboxamide* (**7b**). ν 3456–3378 (NH_2_), 1672 (C=O, amide); ^1^H-NMR: δ 1.30 (t, 3H, CH_3_), 3.82 (q, 2H, CH_2_), 4.44, 6.88 (2s, 4H, 2 NH_2_ exchangeable with D_2_O), 7.32–7.68 (m, 6H, 4 Ph-H + 2 pyr-H), 8.68 (s, 1H, pyr-5'-H); ^13^C-NMR: 13.68, 64.12, 100.00, 102.04, 121.24, 122.12, 127.85, 128.38, 128.55, 134.05, 135.15, 137.05, 146.12, 149.65, 151.00, 154.36, 156.14, 161.42, 164.04; MS, *m/z* (%): 459 [M^+^,25], 287 [100, base peak]; Elemental analysis for C_21_H_16_Cl_2_N_4_O_2_S (459.34): calcd.: C, 54.91; H, 3.51; Cl, 15.44; N, 12.20; S, 6.98. found: C, 54.86; H, 3.45; Cl, 15.39; N, 12.16; S, 6.92.

*3-Amino-6-(2-chloro-6-ethoxypyridin-4-yl)-4-(2,4-dichlorophenyl)thieno[2,3-b]pyridine-2-carboxamide* (**7c**). IR (KBr, cm^−1^): ν 3456 (NH_2_), 1735 (C=O, ester); ^1^H-NMR: δ 1.28 (t, 3H, CH_3_), 3.86 (q, 2H, CH_2_), 4.54, 6.76 (2s, 4H, 2 NH_2_ exchangeable with D_2_O), 7.12–7.73 (m, 5H, 3 Ph-H + 2 pyr-H), 8.48 (s, 1H, pyr-5'-H); ^13^C-NMR: 14.12, 64.33, 101.04, 102.84, 121.32, 122.40, 126.24, 128.65, 128.75, 129.72, 132.32, 135.12, 136.66, 137.22, 145.56, 149.55, 151.22, 154.44, 157.12, 161.26, 164.57; MS, *m/z* (%): 494 [M^+^,12], 320 [100, base peak]; Elemental analysis for C_21_H_15_Cl_3_N4O_2_S (493.79): calcd.: C, 51.08; H, 3.06; Cl, 21.54; N, 11.35; S, 6.49. found: C, 51.00; H, 3.00; Cl, 21.50; N, 11.30; S, 6.44.

*7-(2-Chloro-6-ethoxypyridin-4-yl)-9-(substituted-phenyl)-2-methyl-4H-pyrido[3',2':4,5]thieno[3,2-d]-[1,3]-oxazin-4-ones* **9a**–**c**. A mixture of **6a**–**c** (1 mmol) in ethanolic NaOH (100 mL, 5%) was heated under reflux for 4 h. The solvent was evaporated under reduced pressure, the obtained sodium salt **8a**–**c** was dissolved in acetic anhydride (100 mL) and refluxed it for 6 h. The reaction mixture was concentrated and allowed to cool, poured onto ice water, the obtained solid was collected by filtration, washed with water, dried and crystallized from the proper solvents to afford the corresponding thienooxazinopyridine derivatives **9a**–**c**, respectively. 

*7-(2-Chloro-6-ethoxypyridin-4-yl)-9-(4-fluorophenyl)-2-methyl-4H-pyrido[3',2':4,5]thieno[3,2-d]-[1,3]oxazin-4-one* (**9a**). IR (KBr, cm^−1^): ν 1750 (C=O); ^1^H-NMR: δ 1.30 (t, 3H, CH_3_), 2.03 (s, 3H, CH_3_), 3.78 (q, 2H, CH_2_), 7.04–7.58 (m, 6H, 4 Ph-H + 2 pyr-H), 8.62 (s, 1H, pyr-5'-H); ^13^C-NMR: 14.10, 18.98, 64.30, 100.86, 101.68, 116.02, 120.80, 125.85, 128.42, 132.78, 134.46, 135.35, 145.72, 150.05, 151.75, 154.90, 155.25 158.62, 162.70, 164.08, 165.25; MS, *m/z* (%): 468 [M^+^,6], 217 [100, base peak]; Elemental analysis for C_23_H_15_ClFN_3_O_3_S (467.89): calcd.: C, 59.04; H, 3.23; Cl, 7.58; N, 8.98; S, 6.85. found: C, 58.96; H, 3.18; Cl, 7.52; N, 8.90; S, 6.80.

*7-(2-Chloro-6-ethoxypyridin-4-yl)-9-(4-chlorophenyl)-2-methyl-4H-pyrido[3',2':4,5]thieno[3,2-d]-[1,3]oxazin-4-one* (**9b**). IR (KBr, cm^−1^): ν 1745 (C=O); ^1^H-NMR: δ 1.28 (t, 3H, CH_3_), 2.01 (s, 3H, CH_3_), 3.89 (q, 2H, CH_2_), 7.23–7.65 (m, 6H, 4 Ph-H + 2 pyr-H), 8.42 (s, 1H, pyr-5'-H); ^13^C-NMR: 14.08, 21.60, 64.18, 100.55, 101.45, 121.12, 125.68, 128.12, 128.96, 133.05, 134.58, 135.32, 135.72, 145.92, 149.75, 151.84, 154.86, 155.14, 158.70, 164.12, 165.18; MS, *m/z* (%): 484 [M^+^,15], 156 [100, base peak]; Elemental analysis for C_23_H_15_Cl_2_N_3_O_3_S (484.35): calcd.: C, 57.03; H, 3.12; Cl, 14.64; N, 8.68; S, 6.62. found: C, 56.95; H, 3.10; Cl, 14.60; N, 8.63; S, 6.58.

*7-(2-Chloro-6-ethoxypyridin-4-yl)-9-(2,4-dichlorophenyl)-2-methyl-4H-pyrido[3',2':4,5]thieno[3,2-d]-[1,3]oxazin-4-one* (**9c**). IR (KBr, cm^−1^): ν 1750 (C=O); ^1^H-NMR: δ 1.30 (t, 3H, CH_3_), 2.00 (s, 3H, CH_3_), 3.82 (q, 2H, CH_2_), 7.21–7.68 (m, 5H, 3 Ph-H + 2 pyr-H), 8.54 (s, 1H, pyr-5'-H); ^13^C-NMR: 14.10, 19.18, 64.22, 100.28, 101.15, 121.16, 125.56, 126.46, 128.58, 129.80, 132.44, 134.34, 135.18, 135.45, 136.73, 145.88, 149.72, 151.69, 154.78, 155.18, 159.06, 164.18, 165.32; MS, *m/z* (%): 519 [M^+^,8], 320 [100, base peak]; Elemental analysis for C_23_H_14_Cl_3_N_3_O_3_S (518.79): calcd.: C, 53.25; H, 2.72; Cl, 20.50; N, 8.10; S, 6.18. found: C, 53.18; H, 2.68; Cl, 20.45; N, 8.00; S, 6.12.

*7-(2-Chloro-6-ethoxypyridin-4-yl)-9-(substituted-phenyl)-2-methylpyrido[3',2':4,5]thieno[3,2-d]-pyrimidin-4(3H)-ones* **10a**–**c**

A mixture of **9a**–**c** (1 mmol) and ammonium acetate (0.6 g, 8 mmol) in glacial acetic acid (100 mL) was heated under reflux for 6 h. The reaction mixture was evaporated under reduced pressure, the residue was triturated with cooled water, the solid formed was collected by filtration, washed with water, dried and crystallized from the proper solvents to afford the corresponding thienopyrimidino-pyridine 0.30 g (70%) **10a**–**c**, respectively. 

*7-(2-Chloro-6-ethoxypyridin-4-yl)-9-(4-fluorophenyl)-2-methylpyrido[3',2':4,5]thieno[3,2-d]-pyrimidin-4(3H)-one* (**10a**). IR (KBr, cm^−1^): ν 3420 (NH), 1650 (C=O); ^1^H-NMR: δ 1.28 (t, 3H, CH_3_), 2.32 (s, 3H, CH_3_), 3.86 (q, 2H, CH_2_), 7.12–7.64 (m, 6H, 4 Ph-H + 2 pyr-H), 8.58 (s, 1H, pyr-5'-H), 9.26 (s, 1H, NH exchangeable with D_2_O); ^13^C-NMR: 14.08, 24.98, 64.42, 101.02, 102.54, 116.15, 121.04, 126.14, 128.85, 132.86, 136.76, 137.05, 145.88, 150.15, 151.98, 154.10, 154.86, 157.25, 160.03, 162.99, 164.28; MS, *m/z* (%): 467 [M^+^,18], 156 [100, base peak]; Elemental analysis for C_23_H_16_ClFN_4_O_2_S (466.91): calcd.: C, 59.16; H, 3.45; Cl, 7.59; N, 12.00; S, 6.87. found: C, 59.10; H, 3.38; Cl, 7.52; N, 11.94; S, 6.83.

*7-(2-Chloro-6-ethoxypyridin-4-yl)-9-(4-chlorophenyl)-2-methylpyrido[3',2':4,5]thieno[3,2-d]-pyrimidin-4(3H)-one* (**10b**). IR (KBr, cm^−1^): ν 3438 (NH), 1649 (C=O); ^1^H-NMR: δ 1.31 (t, 3H, CH_3_), 2.24 (s, 3H, CH_3_), 3.78 (q, 2H, CH_2_), 7.33–7.72 (m, 6H, 4 Ph-H + 2 pyr-H), 8.62 (s, 1H, pyr-5'-H), 9.32 (s, 1H, NH exchangeable with D_2_O); ^13^C-NMR: 13.98, 25.04, 64.53, 100.12, 101.36, 121.13, 126.45, 128.15, 129.05, 133.76, 135.99, 136.88, 145.76, 146.05, 149.85, 151.90, 154.16, 154.92, 157.48, 159.73, 164.36; MS, *m/z* (%): 483 [M^+^,18], 326 [100, base peak]; Elemental analysis for C_23_H_16_Cl_2_N_4_O_2_S (483.36): calcd.: C, 57.15; H, 3.34; Cl, 14.67; N, 11.59; S, 6.63. found: C, 57.10; H, 3.28; Cl, 14.62; N, 11.53; S, 6.58.

*7-(2-Chloro-6-ethoxypyridin-4-yl)-9-(2,4-dichlorophenyl)-2-methylpyrido[3',2':4,5]thieno[3,2-d]-pyrimidin-4(3H)-one* (**10c**). IR (KBr, cm^−1^): ν 3465 (NH), 1653 (C=O); ^1^H-NMR: δ 1.26 (t, 3H, CH_3_), 2.12 (s, 3H, CH_3_), 3.80 (q, 2H, CH_2_), 7.21–7.70 (m, 5H, 3 Ph-H + 2 pyr-H), 8.72 (s, 1H, pyr-5'-H), 9.48 (s, 1H, NH exchangeable with D_2_O); ^13^C-NMR: 13.92, 24.87, 64.42, 99.96, 101.02, 120.33, 126.32, 126.64, 128.36, 129.72, 132.88, 135.09, 136.64, 136.84, 145.86, 146.13, 149.77, 151.92, 153.96, 154.66, 157.68, 160.02, 164.48; MS, *m/z* (%): 518 [M^+^,5], 145 [100, base peak]; Elemental analysis for C_23_H_15_Cl_3_N_4_O_2_S (517.81): calcd.: C, 53.35; H, 2.92; Cl, 20.54; N, 10.82; S, 6.19. found: C, 53.30; H, 2.87; Cl, 20.50; N, 10.79; S, 6.14.

*7-(2-Chloro-6-ethoxypyridin-4-yl)-9-(4-fluorophenyl)-2,3-dimethylpyrido[3',2':4,5]thieno[3,2-d]pyrimidin-4(3H)-ones* **11a**–**c**. A solution of **10a**–**c** (1 mmol) in DMF (20 mL) was stirred with anhydrous K_2_CO_3_ (0.19 g, 1 mmol) for 10 min at room temperature, then methyl iodide (0.28 g, 2 mmol) in DMF (5 mL) were added. The reaction mixture was heated at 60 °C for 4 h, after cooling, poured into ice water, and the formed precipitate was filtered off, washed with water, dried and crystallized from the proper solvents to afford the corresponding thieno*-N*-methylpyrimidinopyridines **11a**–**c**, respectively.

*7-(2-Chloro-6-ethoxypyridin-4-yl)-9-(4-fluorophenyl)-2,3-dimethylpyrido[3',2':4,5]thieno[3,2-d]pyrimidin-4(3H)-one* (**11a**). IR (KBr, cm^−1^): ν 1668 (C=O); ^1^H-NMR: δ 1.28 (t, 3H, CH_3_), 2.32, 3.10 (2s, 6H, 2 CH_3_), 3.78 (q, 2H, CH_2_), 7.08–7.68 (m, 6H, 4 Ph-H + 2 pyr-H), 8.62 (s, 1H, pyr-5'-H); ^13^C-NMR: 14.00, 22.14, 26.06, 64.15, 100.10, 101.32, 116.18, 120.34, 126.34, 128.95, 132.90, 136.42, 145.76, 146.15, 149.85, 151.80, 154.02, 154.77, 157.36, 159.63, 162.76, 164.30; MS, *m/z* (%): 481 [M^+^,4], 98 [100, base peak]; Elemental analysis for C_24_H_18_ClFN_4_O_2_S (480.94): calcd.: C, 59.94; H, 3.77; Cl, 7.37; N, 11.65; S, 6.67. found: C, 59.88; H, 3.72; Cl, 7.33; N, 11.60; S, 6.61.

*7-(2-Chloro-6-ethoxypyridin-4-yl)-9-(4-chlorophenyl)-2,3-dimethylpyrido[3',2':4,5]thieno[3,2-d]-pyrimidin-4(3H)-one* (**11b**). IR (KBr, cm^−1^): ν 1670 (C=O); ^1^H-NMR: δ 1.29 (t, 3H, CH_3_), 2.18, 3.06 (2s, 6H, 2 CH_3_), 3.82 (q, 2H, CH_2_), 7.28–7.68 (m, 6H, 4 Ph-H + 2 pyr-H), 8.78 (s, 1H, pyr-5'-H); ^13^C-NMR: 13.86, 22.00, 26.28, 64.14, 99.58, 100.12, 120.56, 126.76, 128.00, 128.95, 133.45, 135.85, 136.56, 145.32, 146.75, 149.80, 150.87, 153.78, 154.65, 157.45, 160.02, 164.28; MS, *m/z* (%): 497 [M^+^,19], 162 [100, base peak]; Elemental analysis for C_24_H_18_Cl_2_N_4_O_2_S (497.39): calcd.: C, 57.95; H, 3.65; Cl, 14.26; N, 11.26; S, 6.45. found: C, 57.90; H, 3.59; Cl, 14.22; N, 11.20; S, 6.40.

*7-(2-Chloro-6-ethoxypyridin-4-yl)-9-(2,4-dichlorophenyl)-2,3-dimethylpyrido[3',2':4,5]thieno[3,2-d]-pyrimidin-4(3H)-one* (**11c**). IR (KBr, cm^−1^): ν 1667 (C=O); ^1^H-NMR: δ 1.28 (t, 3H, CH_3_), 2.22, 2.96 (2s, 6H, 2 CH_3_), 3.80 (q, 2H, CH_2_), 7.24–7.70 (m, 5H, 3 Ph-H + 2 pyr-H), 8.65 (s, 1H, pyr-5'-H); ^13^C-NMR: 13.90, 22.01, 26.48, 64.08, 99.86, 100.09, 120.60, 126.45, 126.58, 128.25, 129.52, 132.66, 135.14, 136.60, 136.78, 145.42, 146.78, 149.82, 151.14, 153.88, 154.72, 157.48, 159.72, 164.20; MS, *m/z* (%): 532 [M^+^,19], 252 [100, base peak]; Elemental analysis for C_24_H_17_Cl_3_N4O_2_S (531.84): calcd.: C, 54.20; H, 3.22; Cl, 20.00; N, 10.53; S, 6.03. found: C, 54.15; H, 3.16; Cl, 19.85; N, 10.48; S, 6.00.

### 3.2. Antimicrobial Screening Media

The following media were used:

1. PDA medium: this medium was used for fungi cultivation. It consists of 4 g dextrose/L potatoes extract.
2. Czapek Dox medium: it consists of 10 g glucose, 2 g KNO_3_, 1g K_2_HPO_4_, 0.5 g KCl, 0.5 g MgSO_4_, and 0.05 g ferrous sulphate/L distilled water. This medium is specialized for bacteria cultivation.
3. Medium 3: it consists of 10 glucose, 5 g peptone, 3 yeast extract, and 3 malt extract. It was used for yeast cultivation.

## 4. Conclusions 

A series of newly compounds **3**–**11** were prepared using citrazinic acid (2,6-dihydroxyisonicotinic acid) as a starting material. The obtained derivatives were screening as antimicrobial and antifungal agents. Four of the synthesized compounds **5a**, **7b**, **9b** and **10b** exhibited potent antibacterial and antifungal bioactivity compared with streptomycin and fusidic acid used as reference drugs. The other tested compounds were found to exhibit moderate to low antibacterial activity. On the other hand when higher concentrations (3× and 4×) of compound **9a**, which exhibited a moderate antibacterial activity, were used, this compound exhibited very good antibacterial activity. While different concentrations of compounds **5a** and **10a** exhibited a very good antifungal activity (2× and 3×).

## Figures and Tables

**Table 1:** Melting points, crystallization solvents, yields, molecular formulae and molecular weights of compounds **3**–**7**.

Comp. No.	X	Y	Yield (%)	M.p. (°C)	Cryst. Solv.	Molecular Formula (Mol. Wt.)
**3a**	F	H	86	185–187	EtOH	C_16_H_13_ClFNO_2_ (505.73)
**3b**	Cl	H	82	155–157	EtOH	C_16_H_13_Cl_2_NO_2_ (322.19)
**3c**	Cl	Cl	85	203–205	EtOH	C_16_H_12_Cl_3_NO_2_ (356.63)
**4a**	F	H	65	192–194	DMF/H_2_O (2:1)	C_19_H_13_ClFN_3_OS (385.84)
**4b**	Cl	H	58	206–208	AcOH/H_2_O (2:1)	C_19_H_13_Cl_2_N_3_OS (402.30)
**4c**	Cl	Cl	70	225–227	DMF/H_2_O (2:1)	C_19_H_12_Cl_3_N_3_OS (436.74)
**5a**	F	H	78	198–200	EtOH/Ether (2:1)	C_23_H_19_ClFN_3_O_3_S (471.93)
**5b**	Cl	H	76	189–191	EtOH/Ether (2:1)	C_23_H_19_Cl_2_N_3_O_3_S (488.39)
**5c**	Cl	Cl	69	245–257	EtOH/Ether (2:1)	C_23_H_18_Cl_3_N_3_O_3_S (522.83)
**6a**	F	H	65	176–178	Dioxane	C_23_H_19_ClFN_3_O_3_S (471.93)
**6b**	Cl	H	70	214–216	EtOH	C_23_H_19_Cl_2_N_3_O_3_S (488.39)
**6c**	Cl	Cl	58	235–237	DMF/EtOH (2:1)	C_23_H_18_Cl_3_N_3_O_3_S (522.83)
**7a**	F	H	86	200–202	MeOH	C_21_H_16_ClFN_4_O_2_S (442.89)
**7b**	Cl	H	85	228–230	AcOH	C_21_H_16_Cl_2_N_4_O_2_S (459.35)
**7c**	Cl	Cl	84	256–258	AcOH/H_2_O (2:1)	C_21_H_15_Cl_3_N_4_O_2_S (493.79)

**Table 2:** Melting points, crystallization solvents, yields, molecular formulae and molecular weights of compounds **9**–**11**.

Comp. No.	X	Y	Yield (%)	M.p. (°C)	Cryst. Solv.	Molecular Formula (Mol. Wt.)
**9a**	F	H	75	195–197	EtOH	C_23_H_15_ClFN_3_O_3_S (467.90)
**9b**	Cl	H	68	214–216	AcOH	C_23_H_15_Cl_2_N_3_O_3_S (484.35)
**9c**	Cl	Cl	60	282–284	DMF/H_2_O (2:1)	C_23_H_14_Cl_3_N_3_O_3_S (518.80)
**10a**	F	H	80	178–180	AcOH/H_2_O (2:1)	C_23_H_16_ClFN_4_O_2_S (466.92)
**10b**	Cl	H	72	188–190	AcOH/H_2_O (2:1)	C_23_H_16_Cl_2_N_4_O_2_S (483.37)
**10c**	Cl	Cl	65	256–258	DMF/H_2_O (2:1)	C_23_H_15_Cl_3_N_4_O_2_S (517.81)
**11a**	F	H	78	186–188	AcOH/H_2_O (2:1)	C_24_H_18_ClFN4O_2_S (480.94)
**11b**	Cl	H	66	200–202	AcOH	C_24_H_18_Cl_2_N_4_O_2_S (497.40)
**11c**	Cl	Cl	72	264–266	DMF/H_2_O (2:1)	C_24_H_17_Cl_3_N_4_O_2_S (531.84)

**Table 3:** Antimicrobial activities of the newly synthesized compounds **3**–**11**.

Comp. No.	Fungi		Yeast		*Str. sp*	Bacteria			
	*A.n*	*Pen. sp*	*C. a*	*R.i*		Gram − ve		Gram + ve	
						*B.s*	*S.l*	*E.c*	*P. sp*
**3a**	12	12	12	11	13	14	14	14	13
**3b**	12	12	10	11	9	8	7	9	11
**3c**	8	10	9	11	12	12	12	11	14
**4a**	10	12	11	11	13	11	10	12	11
**4b**	11	12	13	11	14	13	11	12	12
**4c**	10	12	12	13	13	12	10	12	11
**5a**	17	16	16	17	22	23	24	23	21
**5b**	4	5	4	3	7	8	7	9	8
**5c**	13	12	12	13	11	13	12	10	9
**6a**	10	13	10	11	21	20	21	23	23
**6b**	8	8	6	7	11	12	13	13	12
**6c**	12	13	13	12	13	11	13	12	13
**7a**	13	12	12	13	11	13	12	10	9
**7b**	7	5	8	9	6	12	13	13	12
**7c**	12	13	11	13	12	8	8	6	7
**9a**	12	10	11	11	20	20	21	19	20
**9b**	19	20	19	19	11	13	12	10	9
**9c**	10	11	11	12	10	11	10	12	11
**10a**	15	16	13	14	11	11	12	12	13
**10b**	23	22	22	20	11	23	22	24	23
**10c**	11	10	12	11	11	10	12	11	11
**11a**	13	12	12	13	11	13	12	10	9
**11b**	10	12	11	11	13	11	10	12	11
**11c**	13	11	10	12	11	10	12	11	11
**Streptomycin**	-	-	-	-	21	22	21	22	21	
**Fusidic acid**	17	17	18	18	-	-	-	-	-	

*A.n: Aspergillus niger*; *Pen. sp: Penicillium sp*; *C. a: Candida albican; Str. sp: Streptomyces sp*; *R.i: Rhodotorula ingeniosa*; *B.s: Bacillus subtilis*; *S.l: Streptococcus lactis*; *E.c: Escherichia coli*; *P. sp: Pseudomonas sp.*

**Table 4:** Antibacterial activity of compound **9a** at different concentrations.

Comp. No.	Conc.	*Strep. sp*	Bacteria			
			Gram − ve		Gram + ve	
			*B.s*	*S.l*	*E.c*	*Ps*
**9a**	1×	20	20	21	19	20
	2×	23	23	22	23	22
	3×	25	24	24	24	26
	4×	25	25	27	25	26

Where × = 10 μg.

**Table 5:** Antifungal activity of compounds **5a** and **10a** at different concentrations.

Comp. No.	Conc.	Fungi			
		*A.n*	*Pen. sp*	*C. a*	*R.i*
**5a**	1×	17	16	16	17
	2×	18	18	19	19
	3×	19	20	20	21
	4×	20	22	20	21
**10a**	1×	15	16	13	14
	2×	16	18	18	17
	3×	18	20	20	20
	4×	20	22	20	21

Where × = 10 μg.
